# A *UGT1A1* variant is associated with serum total bilirubin levels, which are causal for hypertension in African-ancestry individuals

**DOI:** 10.1038/s41525-021-00208-6

**Published:** 2021-06-11

**Authors:** Guanjie Chen, Adebowale Adeyemo, Jie Zhou, Ayo P. Doumatey, Amy R. Bentley, Kenneth Ekoru, Daniel Shriner, Charles N. Rotimi

**Affiliations:** Center for Research on Genomics and Global Health, National Human Genome Research Institute, National Institutes of Health, Bethesda, MD USA

**Keywords:** Genetics research, Molecular medicine

## Abstract

Serum bilirubin is associated with several clinical outcomes, including hypertension, type 2 diabetes (T2D), and drug metabolism. Here, we describe findings from our genome-wide association studies (GWAS) of serum (TBIL) using a generalized linear mixed model in West Africans (*n* = 1127), with adjustment for age, sex, body mass index, T2D, significant principal components of population structure, and cryptic relatedness. Genome-wide conditional analysis and CAVIARBF were used to fine map significant loci. The causal effect of TBIL on hypertension was assessed by Mendelian randomization (MR) using the GWAS findings as instrumental variables (IVs) in African Americans (*n* = 3,067). The SNP rs887829 (*UGT1A1*) was significantly associated with TBIL levels (effect allele (*T*) frequency = 0.49, *β* (SE) = 0.59 (0.04), *p* = 9.13 × 10^−54^). Genome-wide conditional analysis and regional fine mapping pointed to rs887829 as a possible causal variant with a posterior inclusion probability of 0.99. The *T* allele of rs887829 is associated with lower hepatic expression of *UGT1A1*. Using rs887829 as an IV, two-stage least-squares MR showed a causal effect of bilirubin on hypertension (*β* = −0.76, 95% CI [−1.52, −0.01], *p* = 0.0459). Our finding confirms that *UGT1A1* influences bilirubin levels. Notably, lower TBIL is causally associated with the increased risk of hypertension.

## Introduction

Hyperbilirubinemia has several causes, including hemolysis, cirrhosis, and bile duct obstruction. In the absence of liver disease, high circulating bilirubin levels have been associated with the reduced risk of several diseases, including respiratory diseases^1^; oxidative stress-mediated diseases, such as diabetes mellitus, diabetic nephropathy, cancer, and cardiovascular disease^2–8^; and hypertension^9^. The protective effect of bilirubin may be due to antioxidant activity^10–13^. There is also evidence that higher bilirubin is associated with the better survival and functional independence in the elderly^14,15^.

The heritability of serum bilirubin has been estimated to be 48 ± 6% (ref. ^16^). The genetic architecture of circulating bilirubin levels has been studied through genome-wide association studies (GWAS), which have revealed several associated variants in one locus, *UGT1A1* (refs. ^17–19^). *UGT1A1* has been found to be a major locus for serum total bilirubin (TBIL) in studies of Han Chinese^20^, individuals with European ancestry^21,22^, and African Americans^23^. The SNP rs887829 in *UGT1A1* accounts for 12% of the variance of TBIL levels in African Americans^23^. UGT1A1, a UDP-glucuronosyltransferase, is the sole enzyme that glucuronidates bilirubin.

Mendelian randomization (MR) is a technique for using genetic variants as instrumental variables (IVs) to estimate the casual effect between exposure and outcome, and has been successfully used in studies of cardiovascular diseases, type 2 diabetes (T2D), heart failure, stroke, and nephrology^19,24–30^. The genetic architecture of common, complex diseases generally consists of common genetic variants with small effect sizes, and it is typically not possible to draw inferences using MR with only a single variant^26^. However, serum metabolites may have simpler genetic architectures^31^, sometimes with a single variant that can be used as a strong IV by itself^27–30^.

Here, we describe a GWAS for serum TBIL in West Africans. We confirm that *UGT1A1* is a major locus for serum TBIL, with conditional analysis revealing only one association signal indexed by rs887829. Using rs887829 as an IV, we demonstrate a causal effect of serum TBIL on hypertension in African Americans, with lower serum (TBIL) being a causal risk factor for hypertension.

## Results

### Population description and genetic architecture

The characteristics of participants in the GWAS and MR studies are presented in Table 1 and Supplementary Table 1, respectively. In all studies, men had a higher mean serum TBIL level than women. Also, in all studies, mean age and prevalence of hypertension was similar for both men and women; however, men had a lower mean body mass index (BMI) compared to women. Overall, the mean age and prevalence of hypertension were higher, and mean serum (TBIL) levels were lower in the sampled West Africans than in the African Americans.

**Table 1:** Study characteristics for genome-wide association study.

	Male^a^	Female	*p* Values
*N* (%)	369 (32.74)	758 (67.26)	4.77E−31
Type 2 diabetes (%)	213 (57.72)	392 (51.72)	0.0577
Hypertension (%)	231 (62.60)	497 (65.57)	0.3286
Age (years)	56.05 (14.03)	55.00 (12.15)	0.2189
BMI (kg/m²)	25.61 (4.48)	30.60 (6.29)	2.92E−39
Bilirubin (mg/dL)	0.62 (0.95)	0.42 (0.27)	1.74E−04

^a^Mean (standard deviation) for continuous variables, *N* (%) for discrete variables.

**Fig. 1:**
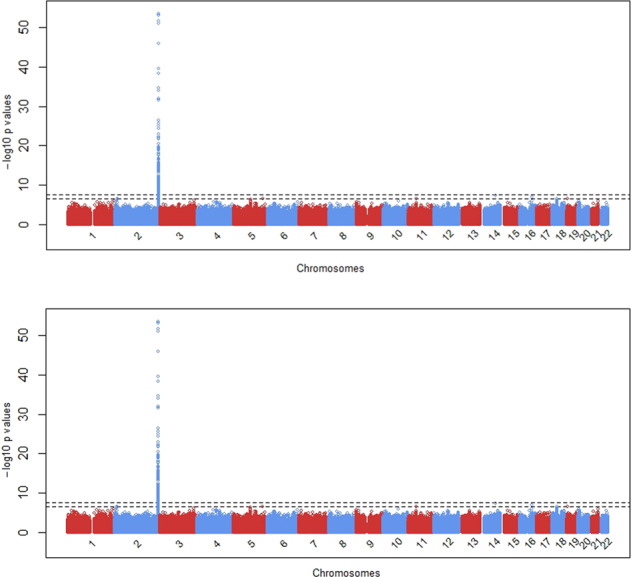
GWAS Manhattan plot. The two dotted lines represent −log_10_ (5 × 10^−8^) and −log_10_ (5 × 10^−7^), respectively.

**Fig. 2:**
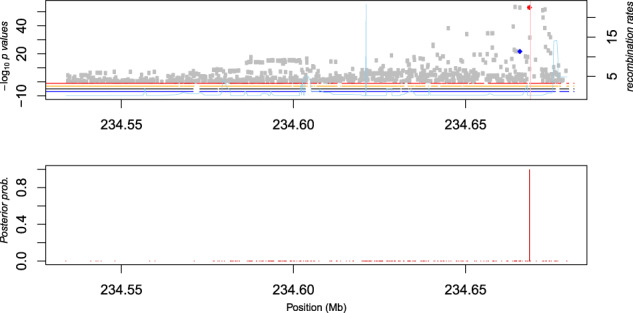
(Top) *UGT1A1* regional association plot. The *x*-axis represents position in Mb and the *y*-axis represents −log_10_ *p* values. Sky-blue lines represent recombination rates (cM/Mb) from the 1000 Genomes Project. LD blocks are shown using horizontal lines: red lines for Africans (AADM), orange for African Americans (HUFS), black for CEU from the 1000 Genomes Project, and blue for CHB from the 1000 Genomes Project. The red dot represents rs887829 and the blue dot represents rs10929302. The pink vertical line represents the position of rs3064744 (not present in these data). (Bottom) posterior inclusion probabilities based on fine mapping. The red vertical line represents the posterior inclusion probability of rs887829.

Heritability for serum TBIL in West Africans was estimated as 0.385 (SD 0.075). Only one locus, centered around *UGT1A1* on chromosome 2, was genome-wide significant (Fig. 1), with the index SNP being rs887829 (*T* allele, effect allele frequency = 0.49, *β* = 0.59, *p* = 9.13 × 10^−54^, Supplementary Table 2). There was no inflation of the test statistic as assessed by a genomic control inflation factor ($\lambda _{\mathrm{GC}}$) of ~1.01 (Supplementary Fig. 1). The *UGT1A1* locus included 205 SNPs (Supplementary Table 2) spanning 419 kb (Fig. 2). Among variants with a minor allele frequency (MAF) of at least 1%, Bayesian fine mapping of the *UGT1A1* locus identified one causal variant, rs887829, with a marginal posterior inclusion probability of 0.99 (Fig. 2). Conditional on rs887829, there was no other genome-wide significant signal (Supplementary Fig. 2).

We next sought to confirm the association of rs887829 with serum TBIL using previously reported GWAS findings in the NHGRI-EBI GWAS Catalog^17,18^. Most previous studies (45/56 or ~80%) identified significant loci in a haplotype block containing rs887829, based on linkage disequilibrium patterns in European ancestry (CEU) and East Asian ancestry (CHB) individuals (Fig. 2 and Supplementary Table 3). This haplotype block was 5.68 kb in West Africans and 5.86 kb in African Americans, compared to 41.80 kb in CEU and CHB.

UGT1A1 is the sole enzyme responsible for the glucuronidation of bilirubin, which occurs in the liver and is necessary for making bilirubin water soluble. We reasoned that if UGT1A1 functions to make bilirubin excretable and hence to lower levels of bilirubin in serum, then the *T* allele of rs887829 should be associated with lower hepatic expression of *UGT1A1*. To test this hypothesis, we interrogated the GTEx database. Consistent with our hypothesis, the *T* allele of rs887829 was associated with lower expression of *UGT1A1* in liver (*p* = 6.3 × 10^−8^).

### Additive Bayesian network

**Fig. 3:**
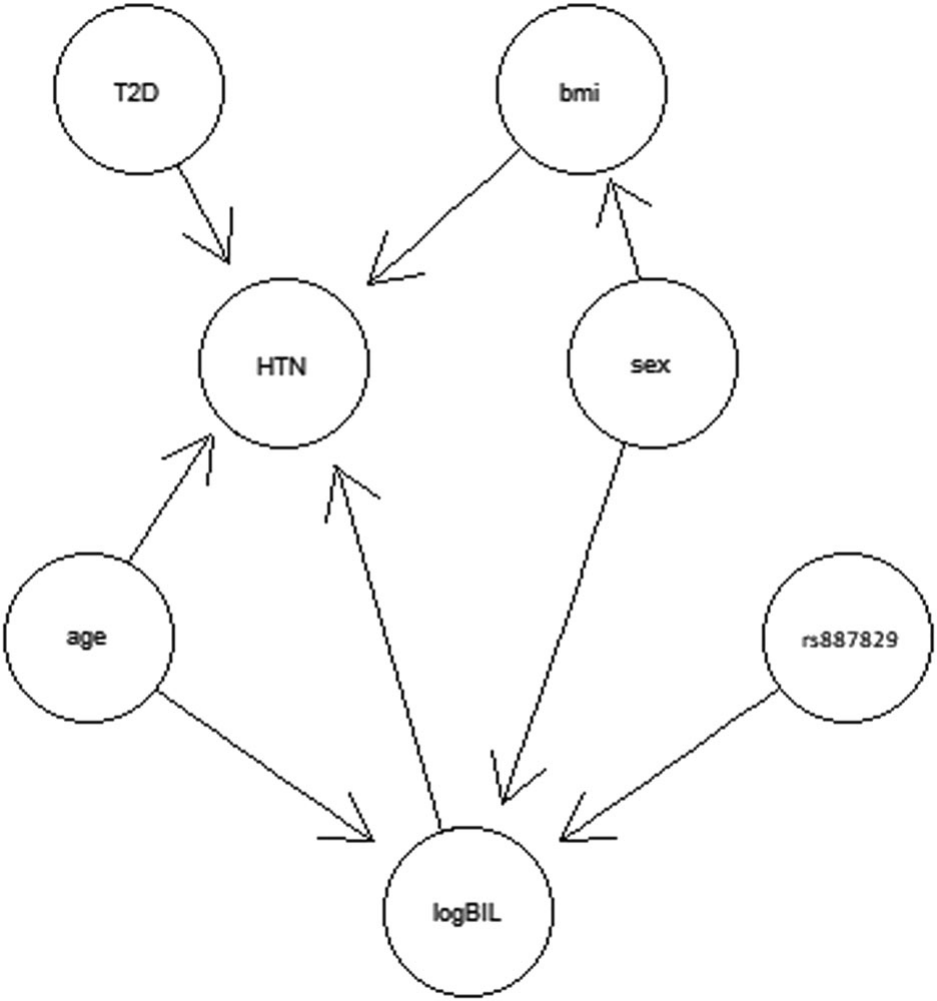
Additive Bayesian network (ABN) plot for African ancestry. The dosage of the genetic variant rs887829 indirectly affects hypertension status via bilirubin levels using 20,000 heuristic searches.

The relationships among rs887829, serum TBIL, hypertension status, sex, age, BMI, and T2D status were evaluated using an additive Bayesian network (ABN) in individuals with African ancestry. Heuristic searching demonstrated that hypertension status was directly influenced by serum TBIL and indirectly influenced by the genetic risk score via serum bilirubin levels (Fig. 3).

### Mendelian randomization

**Fig. 4:**
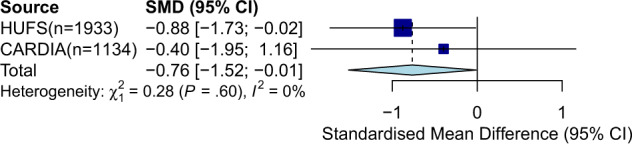
Forest plot of meta-analysis for MR analysis from two studies (HUFS and CARDIA), with a total of 3067 African Americans.

We then performed MR analysis in African Americans to assess the causal effect of serum TBIL on hypertension. To determine whether the MR approach was appropriate, we conducted preliminary analyses to evaluate the following key assumptions. First, there should be a strong association between the IV and the exposure. An *F*-st atistic is used to measure of the strength of the IV, with a threshold of the *F*-statistic > 10 indicating sufficient strength^32^. *F*-statistics of 332.83 and 193.89 were obtained for rs887829 in HUFS and CARDIA, respectively, indicating that rs887829 by itself is a strong IV for serum TBIL. Second, there should be no association between the IV and the outcome, conditional on the exposure. To evaluate this assumption, we regressed hypertension on rs887829, adjusting for serum TBIL as well as age, sex, BMI, and PCs. We observed no effect of rs887829 on hypertension beyond the effect mediated by serum TBIL in HUFS [OR (95% CI) = 1.01 (0.70, 1.73), and *p* = 0.6853] or CARDIA [OR (95% CI) = 1.21 (0.89,1.66), *p* = 0.2294). A third key assumption is that there should be no unmeasured confounders of the association between the IV and the outcome. Although we cannot test for the presence of unmeasured confounders, we included several measured covariates as possible confounders in the regression of prevalent hypertension on rs887829. Finally, we found that higher serum TBIL confers significant protection against prevalent hypertension, with *β* (95% CI) = −0.76 (−1.52, −0.01), and *p* = 0.0459 in combined analysis of African Americans (Fig. 4).

We estimated the conditional power of the MR analysis based on the African American data. Based on a combined sample size *n* = 3067, a significance level *α* = 0.05, a prevalence of hypertension of 0.26, a 0.46 odds ratio of hypertension per standard deviation of serum TBIL, and 12% of the variance of the exposure explained by the IV in African Americans^23^, we estimated a conditional power of 0.99.

## Discussion

*UGT1A1* is known to be a major locus influencing bilirubin levels. In the present study, we confirmed this observation in a study of West Africans and extended the original observation. Taking advantage of the shorter haplotypes and weaker LD in West Africans, we refined the significant region from 10 kb in individuals with European ancestry^33^ to <6 kb.

Previous studies have indicated the functionality of rs887829 or a very close proxy. For example, in a study of infants, the variability of TBIL levels explained by rs887829 increased from 7.0% (day 6) to 10.2% (day 7), consistent with the development of *UGT1A1* isoenzyme expression^34^. The polymorphism rs3064744 is a (TA)_*n*_ tandem repeat covering the TATA box of *UGT1A1* and is a close proxy of rs887829 (*r*^2^ = 0.99)^1,33,35,36^; however, rs3064744 was neither genotyped nor imputed in our study. The SNP rs10929302 (3 kb upstream of rs3064744) is in a phenobarbital response enhancer module and is associated with the response to the anticancer drug irinotecan^37^. Thus, a pleiotropic region (~3 kb, from rs10929302 to rs34983651) influences serum bilirubin levels, phenobarbital induction of *UGT1A1* expression, irinotecan response, and xenobiotic metabolism^23,38^.

The results of ABN modeling suggest that the effect of rs887829 is indirectly associated with the hypertension via an effect mediated by bilirubin. This relationship has been demonstrated experimentally in animal models. Mice treated with indinavir, which induces moderate hyperbilirubinemia by targeting hepatic UGT1A1, had reduced development of hypertension upon Ang II administration compared to mice that did not receive indinavir^39^. The effect was also observed in mice with hyperbilirubinemia, as a result of direct intravenous infusion of bilirubin^39^.

In the present study, we used MR to determine whether serum TBIL causally affects hypertension. Using rs887829 as an IV in a two-stage model, we found evidence of a causal role of serum TBIL on the prevalence of hypertension in African Americans, with lower serum TBIL associated with the increased risk of hypertension. The proportion of variance of the exposure explained by the IVs has a strong effect on the power of MR analysis. One genetic variant, rs887829, explained 12% of the variance of the exposure^23^, so the MR analysis, although based on a single IV, was well powered. In addition, our GWAS was performed on Africans from Ghana and Nigeria, whereas our MR analysis was performed on African Americans. We previously showed that African Americans share ~80% ancestry with West Central Africans, such as Yoruba from Nigeria^40^. To adjust for genetic ancestry, we included principal components in both the GWAS and MR analyses.

The MR analysis indicated that higher serum TBIL is causally associated with a lower risk of hypertension. In mice, bilirubin is a selective ligand for PPARα, driving the expression of genes that result in reduced white adipose tissue size, an increased number of mitochondria, reduced fat accumulation, and reduced insulin resistance^41^. In rats, stimulation of PPARα results in a lowering of blood pressure through increased expression of SOD-1, eNOS, and angiotensin II receptors, implicating protection against hypertension through mechanisms involving antioxidants and nitric oxide^42^. Taken together, these findings suggest that PPARα may be mediating the protective effects of higher serum TBIL on a range of cardiovascular and metabolic diseases.

In summary, rs887829, or a variant in strong LD with it, is likely a causal variant for serum TBIL, possibly via expression of *UGT1A1* in the liver. An association between rs887829 and hypertension is mediated through serum TBIL. In African Americans, lower serum TBIL is causally associated with the risk of hypertension.

## Methods

### Genome-wide association analysis

West African individuals were drawn from the Africa America Diabetes Mellitus (AADM) study^43–46^. We included participants recruited from Ghana or Nigeria. Weight was measured in light clothes on an electronic scale to the nearest 0.1 kg and height was measured with a stadiometer to the nearest 0.1 cm. BMI was computed as weight (kg) divided by the square of height in meters (m^2^). The definition of T2D was based on the American Diabetes Association criteria. Blood samples were drawn after an overnight fast of at least 8 h. Serum TBIL was measured on a COBAS Integra 400 Plus (Roche Diagnostics, Indianapolis, IN, USA) using the Diazo method for a total of 1127 individuals. Participants in the AADM study were genotyped on high-density GWAS arrays (either the Affymetrix® Axiom® Genome-Wide PanAFR Array Set or the Illumina Multi-Ethnic Genotyping Array). After exclusions based on technical quality control (individual call rate ≤ 95%, SNP call rate ≤ 95%, Hardy–Weinberg equilibrium *p* < 10^−6^, and MAF < 0.01), imputation was performed using the African Genome Resources Panel available from the Sanger Imputation Service (https://imputation.sanger.ac.uk/). An info score ≥ 0.3 and MAF ≥ 0.01 were used for filtering imputed variants. Serum TBIL values were regressed on age and sex. The resulting residuals were ranked and inverse normalized. Association analysis was performed using the EPACTS (Efficient and Parallelizable Association Container Toolbox) pipeline (http://genome.sph.umich.edu/wiki/EPACTS), with BMI, T2D status, and significant principal components obtained from the R package SNPRelate^47^ as fixed effects, and genetic cryptic relatedness matrix as a random effect. Based on the Tracy Widom test, three principal components were significant^48^.

### Fine mapping

Fine mapping was performed using the R package CAVIARBF, an approximate Bayesian method that can incorporate functional annotation^49^. Minimal data requirements are marginal statistical test results and linkage disequilibrium between SNPs. SNP annotations were coded for the absence (0) or presence (1) of promoter histone marks, enhancer histone marks, DNAse I hypersensitive sites, or bound proteins as provided by HaploReg v4.1 (https://pubs.broadinstitute.org/mammals/haploreg/haploreg.php). Bayes factors were calculated conditional on a maximum number of causal SNPs. The estimated Bayes factors and prior probabilities were then used to estimate the posterior inclusion probabilities.

### SNP-based heritability

We estimated the heritability of serum TBIL using LDAK^50,51^. A total of 506,737 genotyped SNPs with MAF > 0.05 were used. Log-transformed serum bilirubin levels and covariates of age, sex, BMI, and T2D were used for this estimation. First, based on local LD, we calculated a weight for each predictor (SNP), which showed how well each SNP was tagged. Next, using these weights, we calculated a kinship matrix to improve poorly tagged predictors that had lower than average MAF. Then, we fit the linear mixed model ${\mathrm{Var}}\left( {\mathbf{Y}} \right) = \sigma _g^2{\mathbf{G}} + \sigma _e^2{\mathbf{I}}$, in which **Y** is the vector of phenotype values, **G** is a kinship matrix based on the weighted SNPs, and **I** is an identity matrix. The estimates $\hat \sigma _g^2$ and $\hat \sigma _e^2$ were obtained via restricted maximum likelihood^51^. The proportion of phenotypic variance explained by additive genetic effects at common genotyped variants was estimated as $\hat h_{\mathrm{snp}}^2 = \frac{{\hat \sigma _g^2}}{{\hat \sigma _g^2 + \hat \sigma _e^2}}.$

### Additive Bayesian network modeling

An ABN was used to evaluate relationships among rs887829, hypertension status, and serum TBIL. An ABN is a probabilistic graphic model^52^, extends to the generalized linear model (GLM), and reveals interdependencies among factors which may not be discovered in GLM. In contrast to standard multivariable regression analysis, which only shows interactions between risk factors and the outcome, an ABN illustrates the interactions between all variables. ABN modeling comprises three interrelated parts: parameter learning, network scoring, and structure learning^53^, and were implemented in the R package abn. The standard local heuristic search^54^ was applied to seven nodes (rs887829, bilirubin, hypertension, sex, age, BMI, and T2D) to evaluate node relationships. The majority consensus network was constructed from all edges present in at least 50% of the locally optimal networks found across 20,000 heuristic searches.

### Mendelian randomization analysis

MR was performed to investigate whether serum TBIL has a causal effect on hypertension. Let *Z* represent the genetic variants used as IVs, let *X* represent serum TBIL as the exposure, and let *Y* represent hypertension status as the outcome. Let $\hat X$ represent the values of *X* predicted by *Z*. The causal estimate of the exposure ($\hat X$) on outcome (*Y*), $\beta _{\hat XY}$, can be estimated through two-stage least-squares regression. First, the exposure $\hat X$ is estimated by calculating the fitted values from the regression of *X* on *Z*. Second, $\beta _{\hat XY}$ is obtained by regressing *Y* on $\hat X$. In the data analysis, we first regressed serum TBIL on genetic variants (rs887829, C allele as reference allele) and covariates sex, age, BMI, and PCs to estimate $\hat X$. Second, we regressed hypertension (*Y*) status on $\hat X$ to estimate $\beta _{\hat XY}$, adjusting for sex, age, BMI, and PCs. Analysis was performed using SAS 9.4 (SAS Institute Inc. Cary, NC, USA). Two studies of African Americans, the Howard University Family Study (HUFS, *n* = 1933), and the Coronary Artery Risk Development in Young Adults (CARDIA) study (*n* = 1134), were used for MR analysis. Study procedures in HUFS, including serum TBIL assays and genotyping, have been described previously^23,55^. Participants were not ascertained based on any phenotype. Data from the CARDIA study were retrieved from dbGaP under study accession number phs000285.v3.p2. Meta-analysis with inverse variance weights was performed using the R package meta^56^. Power calculations were performed using mRnd (https://shiny.cnsgenomics.com/mRnd/)^57^.

### Reporting summary

Further information on research design is available in the Nature Research Reporting Summary linked to this article.

## Supplementary information

Reporting Summary

Supplementary Information

## Data Availability

The AADM and HUFS datasets used and/or analyzed in the current study are available from the corresponding author upon reasonable request for collaborative studies that are consistent with the IRB approvals and patient consent. The CARDIA data (phs000285) are available through dbGaP authorized approval.

## Code availability

CAVIARBF is fully available on bitbucket (https://bitbucket.org/Wenan/caviarbf). The bitbucket page describes all dependencies and versions. HaploReg is a fully available on broadinstitute (https://pubs.broadinstitute.org). The broadinstitute web page describes all dependencies and versions. LDAK is a fully available on dougspeed (http://dougspeed.com). The dougspeed web page describes all dependencies and versions. Additive Bayesian network modeling in R is a fully available on http://r-bayesian-networks.org. The web page describes all dependencies and versions. A R package of meta is available on cran (https://cran.r-project.org). The cran web page describes all dependencies and versions. A R package of SNPRelate is available on cran (https://cran.r-project.org). The cran web page describes all dependencies and versions. mRnd is fully available on https://shiny.cnsgenomics.com/mRnd/. The web page describes all dependencies and versions.
