# Supplementary material for: A UGT1A1 variant is associated with serum total bilirubin levels, which are causal for hypertension in African-ancestry individuals
Source: NPJ Genom Med. 2021 Jun 11;6:44. doi: 10.1038/s41525-021-00208-6 (PMC8196001; doi:10.1038/s41525-021-00208-6)
Supplement: Supplementary file 1 — Reporting Summary [file 41525_2021_208_MOESM1_ESM.pdf]

nature research

Corresponding author(s): Dr. Charles Rotimi

Last updated by author(s): 2021/02/08

Reporting Summary

Nature Research wishes to improve the reproducibility of the work that we publish. This form provides structure for consistency and transparency in reporting. For further information on Nature Research policies, see our Editorial Policies and the Editorial Policy Checklist.

Statistics

For all statistical analyses, confirm that the following items are present in the figure legend, table legend, main text, or Methods section.

n/a	Confirmed	
☐	☒	The exact sample size (n) for each experimental group/condition, given as a discrete number and unit of measurement
☒	☐	A statement on whether measurements were taken from distinct samples or whether the same sample was measured repeatedly
☐	☒	The statistical test(s) used AND whether they are one- or two-sided<br>*Only common tests should be described solely by name; describe more complex techniques in the Methods section.*
☐	☒	A description of all covariates tested
☐	☒	A description of any assumptions or corrections, such as tests of normality and adjustment for multiple comparisons
☐	☒	A full description of the statistical parameters including central tendency (e.g. means) or other basic estimates (e.g. regression coefficient) AND variation (e.g. standard deviation) or associated estimates of uncertainty (e.g. confidence intervals)
☐	☒	For null hypothesis testing, the test statistic (e.g. F, t, r) with confidence intervals, effect sizes, degrees of freedom and P value noted<br>*Give P values as exact values whenever suitable.*
☒	☐	For Bayesian analysis, information on the choice of priors and Markov chain Monte Carlo settings
☒	☐	For hierarchical and complex designs, identification of the appropriate level for tests and full reporting of outcomes
☐	☒	Estimates of effect sizes (e.g. Cohen's d, Pearson's r), indicating how they were calculated

Our web collection on statistics for biologists contains articles on many of the points above.

Software and code

Policy information about availability of computer code

Data collection	West African individuals were drawn from the Africa America Diabetes Mellitus (AADM) study. We included participants recruited from Ghana or Nigeria.<br>Two African American studies for MR analysis: the Howard University Family Study (HUFS), and another is from dbGAP [Coronary Artery Risk Development in Young Adults (CARDIA) study].
Data analysis	Statistical packages used in analysis: R 4.0.3 and SAS 9.4

For manuscripts utilizing custom algorithms or software that are central to the research but not yet described in published literature, software must be made available to editors and reviewers. We strongly encourage code deposition in a community repository (e.g. GitHub). See the Nature Research guidelines for submitting code & software for further information.

Data

Policy information about availability of data

All manuscripts must include a data availability statement. This statement should provide the following information, where applicable:

- Accession codes, unique identifiers, or web links for publicly available datasets
- A list of figures that have associated raw data
- A description of any restrictions on data availability

The datasets used and/or analyzed in the current study are available from the corresponding author upon reasonable request. The CARDIA data are deposited in dbGaP and available through dbGaP authorized approval.

Field-specific reporting

Please select the one below that is the best fit for your research. If you are not sure, read the appropriate sections before making your selection.

☒ Life sciences ☐ Behavioural & social sciences ☐ Ecological, evolutionary & environmental sciences

For a reference copy of the document with all sections, see nature.com/documents/nr-reporting-summary-flat.pdf

Life sciences study design

All studies must disclose on these points even when the disclosure is negative.

Sample size	West Africans (n = 1,127)
Data exclusions	Individuals without both genotype and phenotype data
Replication	African Americans from HUFS (n = 1,933) and CARDIA (n = 1,134)
Randomization	All studies were observational, not experimental; specified covariates were included in all regression analyses
Blinding	Blinding was not necessary because no study was a randomized clinical trial

Reporting for specific materials, systems and methods

We require information from authors about some types of materials, experimental systems and methods used in many studies. Here, indicate whether each material, system or method listed is relevant to your study. If you are not sure if a list item applies to your research, read the appropriate section before selecting a response.

Materials & experimental systems

n/a	Involved in the study	
☒	☐	Antibodies
☒	☐	Eukaryotic cell lines
☒	☐	Palaeontology and archaeology
☒	☐	Animals and other organisms
☐	☒	Human research participants
☒	☐	Clinical data
☒	☐	Dual use research of concern

Methods

n/a	Involved in the study	
☒	☐	ChIP-seq
☒	☐	Flow cytometry
☒	☐	MRI-based neuroimaging

Human research participants

Policy information about studies involving human research participants

Population characteristics	West African study individuals were drawn from the Africa America Diabetes Mellitus study (AADM), a large, ongoing genetic epidemiology study including cases and controls for type 2 diabetes. Participants include unrelated and related men and women. The Howard University Family Study (HUFS) is a cross-sectional study of African Americans of the Washington, DC metropolitan area, not ascertained on any phenotype. CARDIA is a prospective longitudinal cohort of healthy black and white adults aged 18-30 at baseline, selected to have approximately the same number of people in subgroups of age, sex, race, and education within each of the field centers (Birmingham, AL; Chicago, IL; Minneapolis, MN; and Oakland, CA).
Recruitment	West Africans: study individuals were drawn from a large, ongoing genetic epidemiology study of type 2 diabetes (T2D) and related traits, the Africa America Diabetes Mellitus study. Cases and controls were recruited through study research clinics at five centers in West Africa. African Americans from the Howard University Study were a consecutive sample of families and unrelated African Americans from the Washington, DC metropolitan area enrolled through door-to-door canvassing, advertisements in local print media, and at community gatherings and focused on the Washington, DC wards that were majority African Americans. CARDIA participants were recruited by random-digit dialing from total communities or specific census tracts or randomly selected from a health-care plan. For this study, data were accessed through dbGAP (study accession number phs000285.v3.p2).
Ethics oversight	AADM: the IRB of the National Institutes of Health; HUFS: the Howard University IRB; our use of dbGAP datasets (CARDIA): the IRB of the National Institutes of Health

Note that full information on the approval of the study protocol must also be provided in the manuscript.
