# Supplementary material for: A UGT1A1 variant is associated with serum total bilirubin levels, which are causal for hypertension in African-ancestry individuals
Source: NPJ Genom Med. 2021 Jun 11;6:44. doi: 10.1038/s41525-021-00208-6 (PMC8196001; doi:10.1038/s41525-021-00208-6)
Supplement: Supplementary file 2 — Supplementary Information [file 41525_2021_208_MOESM2_ESM.pdf]

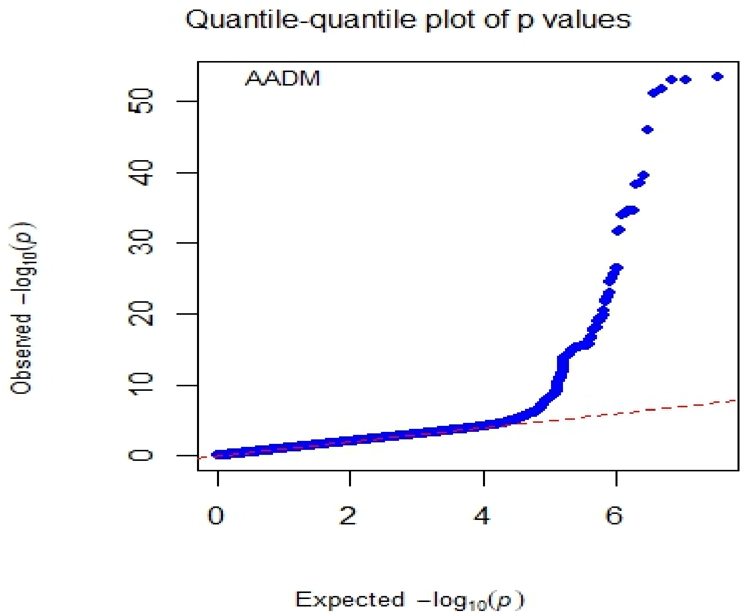

Supplementary Figure 1: Quantile-quantile plot for serum total bilirubin levels. The x-axis represents expected *p*-values and the y-axis represents observed *p*-values.

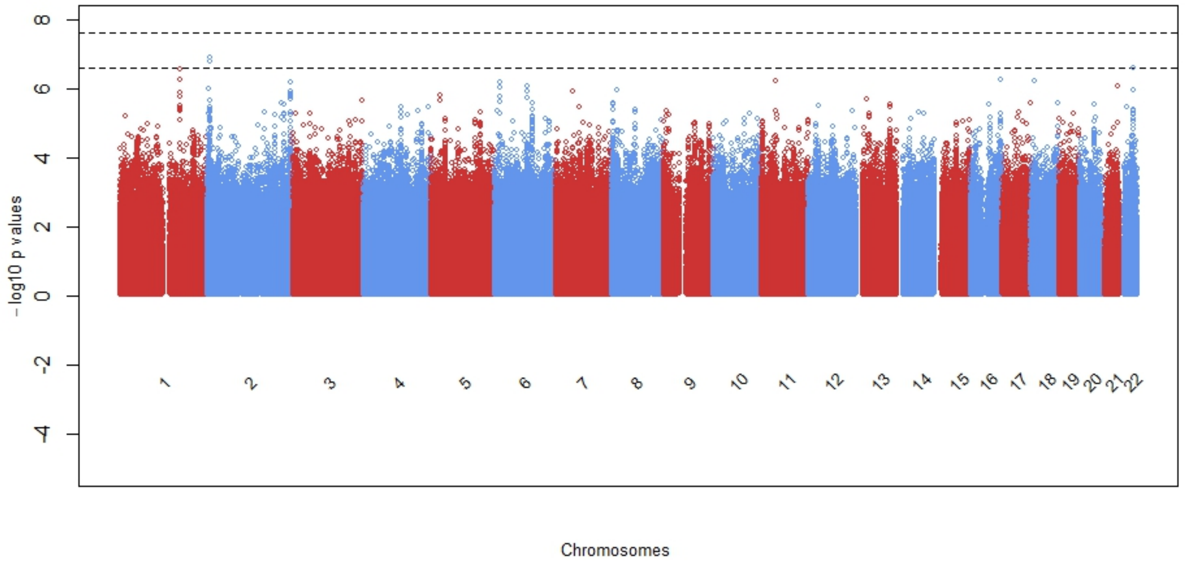

Supplementary Figure 2: Manhattan plot for West Africans, conditional on rs887829. The two dotted lines represent $-\log_{10}(5\times10^{-8})$ and $-\log_{10}(5\times10^{-7})$, respectively.

Supplementary Table 1: Study characteristics for Mendelian randomization (MR) study

	African Americans (HUFS)			African Americans (CARDIA)		
	Male	Female	*P*-values	Male	Female	p -values
N (%)	745 (38.56)	1,188 (61.46)	7.05E-24	468 (41.27)	666 (58.73)	4.11E-09
Hypertension (%) **	260 (34.90)	437 (36.78)	0.4009	39 (3.44)	79 (6.97)	0.0554
Age (Years)	38.62 (16.38)	39.64 (16.65)	0.1866	24.40 (3.73)	24.48 (3.91)	0.7171
BMI (kg/m²)	27.94 (7.44)	31.21 (9.07)	2.93E-16	24.43 (4.20)	25.80 (6.08)	8.58E-06
Bilirubin (mg/dL)	0.71 (0.40)	0.51 (0.27)	2.20E-33	0.68 (0.54)	0.45 (0.24)	1.07E-21

* Mean (SD) for continuous variables, N (%) for discrete variables

** N(%) number of case and percentage by gender

Supplementary Table 2: GWAS results in Africans

Chr	POS	SNP	N	ALT	REF	MAF	Beta	SE	P values
2	234664586	rs35754645	1127	A	ATC	0.4889	0.5960	0.0384	3.21E-54
2	234668570	rs887829	1127	T	C	0.4858	0.5944	0.0385	9.13E-54
2	234665983	rs1976391	1127	A	G	0.4853	-0.5949	0.0385	9.38E-54
2	234673309	2:234673309	1127	T	C	0.4853	0.5887	0.0387	2.20E-52
2	234672639	rs6742078	1127	T	G	0.4737	0.5891	0.0389	8.53E-52
2	234672722	rs4148324	1127	T	G	0.4912	-0.5575	0.0389	1.16E-46
2	234664060	rs11673726	1127	T	G	0.4371	0.5338	0.0402	3.13E-40
2	234673239	rs3771341	1127	A	G	0.4362	0.5289	0.0404	3.83E-39
2	234645186	rs7564935	1127	T	G	0.4358	0.5235	0.0401	4.79E-39
2	234658116	rs7604115	1127	T	C	0.4523	0.4899	0.0394	2.01E-35
2	234656640	rs11888459	1127	T	C	0.4500	-0.4902	0.0395	2.15E-35
2	234657877	rs10178992	1127	A	T	0.4500	0.4902	0.0395	2.15E-35
2	234650562	rs34352510	1127	T	C	0.4478	-0.4823	0.0392	8.18E-35
2	234674476	rs929596	1127	A	G	0.4666	-0.4995	0.0406	1.02E-34
2	234674903	rs67299519	1127	A	AGTGGCCCGGGCTC G	0.4866	0.4847	0.0408	1.32E-32
2	234668879	rs34983651	1127	CAT	C	0.4247	0.4774	0.0403	2.55E-32
2	234640782	2:234640782	1127	G	GTTTGT	0.5151	0.4381	0.0406	3.46E-27
2	234640783	2:234640783	1127	T	TTTG	0.5151	0.4381	0.0406	3.46E-27
2	234640775	rs201829156	1127	G	GTTGT	0.5419	0.4384	0.0413	2.44E-26
2	234673462	rs4148326	1127	T	C	0.6303	-0.4364	0.0417	1.14E-25
2	234657983	rs10179091	1127	T	C	0.6080	-0.4195	0.0405	3.74E-25
2	234664354	rs6747843	1127	A	G	0.3368	0.4353	0.0434	1.24E-23
2	234674252	rs4663971	1127	C	G	0.6628	-0.4269	0.0431	4.15E-23
2	234664765	rs6714634	1127	T	C	0.3395	-0.4273	0.0437	1.28E-22
2	234665782	rs10929302	1127	A	G	0.3395	0.4239	0.0435	1.90E-22
2	234674920	2:234674920	1127	G	GGCCCGGGCTCGGT	0.4203	0.3980	0.0421	3.01E-21
2	234656517	rs12466997	1127	T	C	0.3220	0.4021	0.0433	1.74E-20
2	234654449	rs11695484	1127	A	G	0.3462	-0.3946	0.0429	3.29E-20
2	234652717	rs17864701	1127	T	C	0.3471	0.3926	0.0427	3.69E-20
2	234642015	rs13410335	1127	T	G	0.4924	-0.3822	0.0417	4.95E-20
2	234659061	rs2885296	1127	A	C	0.3475	-0.3890	0.0428	9.59E-20
2	234628679	rs13401281	1127	T	G	0.5963	-0.3812	0.0420	1.20E-19
2	234643560	rs7576166	1127	A	G	0.7392	-0.4216	0.0475	6.46E-19
2	234643737	rs4477910	1127	A	T	0.7392	-0.4216	0.0475	6.46E-19
2	234667582	rs3755319	1127	A	C	0.7502	-0.4275	0.0482	7.27E-19
2	234668245	rs759174	1127	A	C	0.7498	-0.4233	0.0482	1.68E-18
2	234635241	rs6715325	1127	T	C	0.5882	-0.3725	0.0425	2.01E-18
2	234587848	rs75444879	1127	A	G	0.3683	0.3654	0.0418	2.15E-18
2	234637022	rs1983023	1127	T	C	0.7436	-0.4082	0.0480	1.77E-17
2	234642838	rs7567468	1127	T	C	0.3083	0.3844	0.0453	2.07E-17

2	234604068	rs572259405	1127	CCTT	C	0.2983	-0.3763	0.0444	2.39E-17
2	234663649	rs10929301	1127	C	G	0.7399	-0.4008	0.0477	4.17E-17
2	234637912	rs6431625	1127	T	C	0.7432	-0.4031	0.0480	4.74E-17
2	234670560	rs28900396	1127	T	C	0.2672	0.3912	0.0474	1.55E-16
2	234635367	rs17864697	1127	T	C	0.1949	0.4323	0.0526	2.09E-16
2	234655313	rs4663969	1127	A	C	0.5593	0.3321	0.0405	2.45E-16
2	234658623	rs2221198	1127	A	G	0.5593	0.3321	0.0405	2.45E-16
2	234658250	rs7556676	1127	A	G	0.5611	-0.3325	0.0406	2.48E-16
2	234600335	rs45549435	1127	CAGGAG	C	0.4104	-0.3374	0.0413	2.89E-16
2	234600274	rs10445704	1127	A	G	0.4090	0.3385	0.0414	2.99E-16
2	234599296	rs45615240	1127	T	C	0.4108	-0.3374	0.0413	3.02E-16
2	234598991	rs7563561	1127	T	G	0.4059	-0.3375	0.0413	3.04E-16
2	234597483	rs11680450	1127	T	C	0.4095	-0.3385	0.0414	3.12E-16
2	234597667	rs10171367	1127	C	G	0.4095	-0.3385	0.0414	3.12E-16
2	234594083	rs4553819	1127	A	G	0.3957	-0.3420	0.0419	3.23E-16
2	234648860	rs2018985	1127	A	G	0.7824	-0.3960	0.0485	3.33E-16
2	234601965	rs1105880	1127	A	G	0.4033	-0.3405	0.0418	3.48E-16
2	234624286	rs6749496	1127	T	C	0.7209	-0.3720	0.0458	4.80E-16
2	234599089	rs7608175	1127	C	G	0.3921	-0.3389	0.0418	4.95E-16
2	234593117	rs4261716	1127	T	G	0.3961	0.3402	0.0419	4.97E-16
2	234593706	rs13002774	1127	A	G	0.3961	0.3402	0.0419	4.97E-16
2	234593931	rs4338954	1127	C	G	0.3961	-0.3402	0.0419	4.97E-16
2	234594269	rs11902131	1127	T	C	0.3961	0.3402	0.0419	4.97E-16
2	234596865	rs10175809	1127	A	T	0.3961	0.3402	0.0419	4.97E-16
2	234596988	rs10168333	1127	T	C	0.3961	0.3402	0.0419	4.97E-16
2	234599941	rs12623271	1127	C	G	0.4108	-0.3341	0.0412	5.42E-16
2	234592816	rs6724485	1127	A	G	0.3957	0.3393	0.0419	5.71E-16
2	234593041	rs4347832	1127	T	C	0.3957	-0.3393	0.0419	5.71E-16
2	234601669	rs6759892	1127	T	G	0.4095	-0.3356	0.0415	5.80E-16
2	234596836	rs10168155	1127	T	C	0.4095	0.3353	0.0415	6.74E-16
2	234595615	rs6753320	1127	A	C	0.4090	-0.3353	0.0415	6.76E-16
2	234595747	rs6736508	1127	A	G	0.4090	0.3353	0.0415	6.76E-16
2	234595817	rs6753569	1127	A	C	0.4090	-0.3353	0.0415	6.76E-16
2	234595950	rs6736743	1127	A	G	0.4090	0.3353	0.0415	6.76E-16
2	234633345	rs138869941	1127	T	C	0.6142	0.3426	0.0424	6.85E-16
2	234596368	rs10203266	1127	C	G	0.3966	0.3362	0.0419	1.01E-15
2	234600999	rs13015720	1127	A	G	0.4081	0.3319	0.0415	1.25E-15
2	234633242	rs202203863	1127	C	G	0.6182	-0.3399	0.0426	1.50E-15
2	234587847	rs77070100	1127	T	C	0.4784	-0.3133	0.0396	2.44E-15
2	234587852	rs35984508	1127	A	G	0.4784	0.3133	0.0396	2.44E-15
2	234611094	rs17863787	1127	T	G	0.2855	-0.3598	0.0456	2.83E-15
2	234649302	rs17862875	1127	A	G	0.3119	0.3439	0.0436	3.04E-15
2	234667937	rs2003569	1127	A	G	0.2640	-0.3746	0.0476	3.82E-15
2	234661948	rs17862878	1127	A	G	0.2671	-0.3727	0.0475	4.17E-15

2	234587709	rs10207520	1127	A	C	0.2538	-0.3629	0.0463	4.58E-15
2	234612453	rs34781889	1127	CTCTG	C	0.2855	-0.3560	0.0455	4.92E-15
2	234597825	rs10179094	1127	A	T	0.2693	0.3597	0.0461	5.80E-15
2	234587707	rs10202865	1127	T	C	0.2529	0.3610	0.0463	6.03E-15
2	234587208	rs11692664	1127	A	G	0.2547	-0.3604	0.0463	6.72E-15
2	234590041	rs4530361	1127	A	G	0.2693	-0.3583	0.0461	7.63E-15
2	234589190	rs10197460	1127	T	G	0.2689	0.3579	0.0461	8.26E-15
2	234589312	rs10167119	1127	T	C	0.2689	-0.3579	0.0461	8.26E-15
2	234602202	rs1105879	1127	A	C	0.3310	-0.3365	0.0434	8.67E-15
2	234645156	rs77531777	1127	T	C	0.2591	0.3752	0.0485	9.87E-15
2	234590527	rs7586110	1127	T	G	0.2689	-0.3561	0.0462	1.30E-14
2	234590616	rs7577677	1127	A	C	0.2689	0.3561	0.0462	1.30E-14
2	234591205	rs11692021	1127	T	C	0.2689	-0.3561	0.0462	1.30E-14
2	234602191	rs2070959	1127	A	G	0.2618	-0.3576	0.0465	1.40E-14
2	234656032	rs28900380	1127	A	G	0.2940	-0.3495	0.0455	1.63E-14
2	234604078	2:234604078	1127	CTTCT	C	0.2801	-0.3452	0.0450	1.73E-14
2	234604079	2:234604079	1127	T	TTC	0.2801	0.3452	0.0450	1.73E-14
2	234586574	rs7571915	1127	A	G	0.2574	0.3527	0.0463	2.58E-14
2	234597087	2:234597087	1127	C	G	0.2587	-0.3477	0.0466	8.19E-14
2	234597321	rs10173355	1127	A	T	0.2605	-0.3464	0.0465	9.19E-14
2	234633837	rs191532024	1127	C	G	0.3200	-0.3154	0.0434	3.90E-13
2	234633652	rs112132688	1127	A	G	0.3150	0.3233	0.0451	7.12E-13
2	234639310	rs11891311	1127	A	G	0.6365	0.3124	0.0439	1.10E-12
2	234677225	2:234677225	1127	CTTCTTTTTTT	C	0.1259	0.4372	0.0621	1.89E-12
2	234626287	2:234626287	1127	A	G	0.7994	0.3536	0.0515	6.30E-12
2	234605835	rs1604144	1127	T	C	0.4667	-0.2851	0.0417	8.51E-12
2	234637571	rs573079837	1127	G	GAAA	0.1500	0.4034	0.0595	1.17E-11
2	234637573	rs547453547	1127	A	T	0.1500	-0.4034	0.0595	1.17E-11
2	234637575	rs533311965	1127	T	G	0.1500	-0.4034	0.0595	1.17E-11
2	234637578	rs535307581	1127	C	G	0.1500	0.4034	0.0595	1.17E-11
2	234637579	2:234637579	1127	T	G	0.1500	0.4034	0.0595	1.17E-11
2	234637580	rs566067568	1127	T	G	0.1500	0.4034	0.0595	1.17E-11
2	234637581	rs534776981	1127	T	C	0.1500	0.4034	0.0595	1.17E-11
2	234637582	rs557769938	1127	T	C	0.1500	0.4034	0.0595	1.17E-11
2	234664268	rs28900393	1127	A	G	0.1468	-0.4114	0.0608	1.36E-11
2	234637572	2:234637572	1127	A	AAAAACTG	0.1473	0.4020	0.0599	1.99E-11
2	234660486	rs55891750	1127	T	C	0.1726	0.3642	0.0546	2.55E-11
2	234639168	rs45560734	1127	A	G	0.1522	-0.3966	0.0596	2.88E-11
2	234675829	rs34650714	1127	T	C	0.1388	-0.4120	0.0621	3.15E-11
2	234606162	rs6715829	1127	A	T	0.5053	-0.2742	0.0416	4.20E-11
2	234637574	rs545859432	1127	C	G	0.1468	0.3913	0.0598	6.16E-11
2	234637577	rs569892277	1127	T	C	0.1468	0.3913	0.0598	6.16E-11
2	234641908	rs11685892	1127	A	T	0.7913	0.3353	0.0521	1.18E-10
2	234647317	rs6722076	1127	A	G	0.2173	0.3219	0.0507	2.22E-10

2	234625622	rs1875263	1127	T	C	0.6830	0.2796	0.0445	3.30E-10
2	234617407	rs7583278	1127	T	C	0.6811	0.2751	0.0444	5.79E-10
2	234668828	rs34547608	1127	T	C	0.0960	0.4481	0.0724	6.08E-10
2	234648452	rs11695770	1127	T	C	0.3542	0.2741	0.0446	8.11E-10
2	234578020	rs77668415	1127	T	G	0.1286	-0.3902	0.0639	1.05E-09
2	234625936	rs3806592	1127	T	C	0.6727	0.2693	0.0442	1.12E-09
2	234604230	rs28899170	1127	A	C	0.3081	0.2777	0.0457	1.21E-09
2	234672338	rs28900398	1127	T	G	0.0964	0.4389	0.0724	1.34E-09
2	234579204	rs139582139	1127	CT	C	0.1299	0.3831	0.0638	1.91E-09
2	234673510	rs147410587	1127	T	TGAAAAGG	0.1423	0.3568	0.0594	1.92E-09
2	234673512	2:234673512	1127	A	AGCC	0.1423	-0.3568	0.0594	1.92E-09
2	234673516	rs17868342	1127	A	C	0.1423	-0.3568	0.0594	1.92E-09
2	234662173	rs28900387	1127	T	C	0.0946	0.4386	0.0732	2.07E-09
2	234633945	rs59772713	1127	C	G	0.7506	-0.2507	0.0420	2.47E-09
2	234633951	rs59627078	1127	C	G	0.7506	-0.2507	0.0420	2.47E-09
2	234633957	rs57258852	1127	C	G	0.7506	-0.2507	0.0420	2.47E-09
2	234577013	rs28970003	1127	A	G	0.1295	-0.3802	0.0639	2.64E-09
2	234578439	rs28970007	1127	T	C	0.1295	-0.3802	0.0639	2.64E-09
2	234662308	rs28900388	1127	T	C	0.0967	-0.4145	0.0697	2.77E-09
2	234628529	rs871514	1127	T	C	0.8404	-0.3446	0.0583	3.40E-09
2	234652640	rs60469444	1127	A	T	0.0964	-0.4273	0.0724	3.60E-09
2	234641139	rs141356640	1127	A	G	0.0933	-0.4344	0.0737	3.78E-09
2	234642767	rs76091685	1127	A	T	0.0933	-0.4344	0.0737	3.78E-09
2	234645760	rs79657348	1127	A	T	0.0933	-0.4344	0.0737	3.78E-09
2	234684033	rs115951895	1127	T	C	0.1013	0.4226	0.0717	3.85E-09
2	234684188	rs115692048	1127	T	G	0.1013	0.4226	0.0717	3.85E-09
2	234620171	rs28898593	1127	A	G	0.0906	0.4376	0.0744	4.01E-09
2	234621436	rs28898600	1127	T	G	0.0906	0.4376	0.0744	4.01E-09
2	234689830	rs9287649	1127	C	G	0.1280	-0.3701	0.0631	4.53E-09
2	234673514	rs62191921	1127	C	G	0.1400	0.3513	0.0600	4.66E-09
2	234673515	rs17864707	1127	C	G	0.1400	0.3513	0.0600	4.66E-09
2	234679974	2:234679974	1127	C	G	0.3997	0.2548	0.0435	4.72E-09
2	234633963	rs61475847	1127	T	C	0.7524	0.2466	0.0421	4.89E-09
2	234628526	rs28898611	1127	C	G	0.0928	-0.4308	0.0738	5.18E-09
2	234630611	rs58524075	1127	A	G	0.0928	0.4308	0.0738	5.18E-09
2	234625297	rs6744284	1127	T	C	0.6624	0.2560	0.0439	5.62E-09
2	234672663	rs3771342	1127	T	G	0.1181	-0.3651	0.0627	5.85E-09
2	234692189	2:234692189	1127	G	GTATT	0.1306	-0.3633	0.0626	6.63E-09
2	234649573	2:234649573	1127	G	GT	0.8801	0.3635	0.0628	7.12E-09
2	234650193	rs4663963	1127	T	G	0.8801	-0.3635	0.0628	7.12E-09
2	234584306	rs2602380	1127	T	C	0.6324	-0.2519	0.0435	7.23E-09
2	234687118	rs34670649	1127	T	C	0.1018	-0.4127	0.0717	8.51E-09
2	234279209	2:234279209	1127	A	ACTT	0.4483	0.2451	0.0427	9.19E-09
2	234632130	rs6750992	1127	A	C	0.8476	0.3254	0.0567	9.80E-09

2	234644421	rs2363116	1127	C	G	0.8431	-0.3365	0.0587	1.02E-08
2	234583490	rs10189426	1127	T	C	0.1668	0.3172	0.0555	1.12E-08
2	234583846	rs12615708	1127	A	G	0.1668	0.3172	0.0555	1.12E-08
2	234635467	rs4294999	1127	A	G	0.8435	-0.3356	0.0588	1.14E-08
2	234637853	rs6706232	1127	A	G	0.8435	0.3356	0.0588	1.14E-08
2	234638249	rs7574296	1127	A	G	0.8435	-0.3356	0.0588	1.14E-08
2	234627992	2:234627992	1127	A	T	0.0875	0.4347	0.0762	1.17E-08
2	234605251	rs112723588	1127	CCTTT	C	0.3811	0.2437	0.0427	1.19E-08
2	234604903	rs6751673	1127	A	G	0.5184	-0.2361	0.0414	1.19E-08
2	234581834	rs2741049	1127	T	C	0.6324	-0.2485	0.0436	1.22E-08
2	234582212	rs2602377	1127	A	G	0.6324	0.2485	0.0436	1.22E-08
2	234582504	rs2602378	1127	A	T	0.6324	-0.2485	0.0436	1.22E-08
2	234583183	rs2741050	1127	A	T	0.6324	0.2485	0.0436	1.22E-08
2	234679384	rs11563251	1127	T	C	0.4050	-0.2488	0.0437	1.27E-08
2	234634916	rs6711351	1127	A	G	0.8458	-0.3373	0.0593	1.28E-08
2	234593374	rs4583459	1127	T	G	0.5891	0.2361	0.0415	1.29E-08
2	234631921	rs6760025	1127	T	C	0.8275	-0.3108	0.0548	1.41E-08
2	234571117	rs28969998	1127	T	G	0.1187	0.3733	0.0664	1.87E-08
2	234633843	rs183860264	1127	C	G	0.2553	-0.2583	0.0461	2.09E-08
2	234583062	rs2602379	1127	A	G	0.6324	0.2443	0.0437	2.18E-08
2	234581654	rs2741047	1127	A	T	0.6310	-0.2431	0.0435	2.22E-08
2	234581748	rs2741048	1127	A	C	0.6310	0.2431	0.0435	2.22E-08
2	234607394	rs12988520	1127	A	C	0.4308	-0.2347	0.0420	2.33E-08
2	234622429	rs2012734	1127	T	C	0.8315	-0.3147	0.0567	2.92E-08
2	234579892	rs3806598	1127	A	C	0.1494	-0.3226	0.0583	3.14E-08
2	234696102	rs10580249	1127	T	TTA	0.2404	0.2759	0.0499	3.15E-08
2	234647944	rs11691827	1127	T	C	0.3114	-0.2583	0.0470	3.86E-08
2	234698097	rs12468017	1127	T	C	0.1958	-0.2889	0.0527	4.32E-08
2	234651800	2:234651800	1127	T	C	0.1057	0.3620	0.0661	4.44E-08
2	234596421	rs28899168	1127	A	G	0.1099	0.3705	0.0678	4.52E-08
2	234692185	rs182606146	1127	A	C	0.1000	-0.3916	0.0717	4.64E-08
2	234581920	rs7349250	1127	A	G	0.1627	-0.3058	0.0560	4.81E-08
2	234664644	rs13403585	1127	T	G	0.0927	-0.3870	0.0710	4.94E-08

Supplementary Table 3: Associated UGT1A variants from previously reported GWAS studies of bilirubin.

DATE ADDED TO CATALOG	PUBMED ID	INITIAL SAMPLE SIZE	Ancestry of population	MAPPED_GENE	SNPS	POS	CONTEXT	*P*-VALUE
5/12/2009	19414484	9,464 individuals	CEU	*UGT1A6, UGT1A8, UGT1A1, UGT1A3, UGT1A9, UGT1A4, UGT1A5, UGT1A7, UGT1A10*	rs6742078	234672639	intron_variant	5E-324
5/19/2009	19419973	4,300 Sardinian individuals	CEU	*UGT1A6, UGT1A8, UGT1A7, UGT1A4, UGT1A10, UGT1A3, UGT1A9, UGT1A5*	rs887829	234668570	intron_variant	1.00E-69
11/16/2013	20639394	8,841 Korean ancestry individuals	CHB	*UGT1A10, UGT1A6, UGT1A4, UGT1A8, UGT1A3, UGT1A1, UGT1A9, UGT1A7, UGT1A5*	rs4148323	234669144	missense_variant	3.00E-139
11/16/2013	20639394	8,841 Korean ancestry individuals	CHB	*UGT1A6, UGT1A8, UGT1A1, UGT1A3, UGT1A9, UGT1A4, UGT1A5, UGT1A7, UGT1A10*	rs6742078	234672639	intron_variant	1.00E-158
6/20/2011	21646302	6,307 European ancestry individuals	CEU	*UGT1A7, UGT1A6, UGT1A9, UGT1A1, UGT1A4, UGT1A10, UGT1A8, UGT1A5, UGT1A3*	rs4148325	234673309	intron_variant	5.00E-62
12/17/2011	22085899	619 African American individuals	YRI	*UGT1A6, UGT1A8, UGT1A7, UGT1A4, UGT1A10, UGT1A3, UGT1A9, UGT1A5*	rs887829	234668570	intron_variant	2.00E-22
6/22/2012	22558097	905 African American cases	YRI	*UGT1A6, UGT1A8, UGT1A7, UGT1A4, UGT1A10, UGT1A3, UGT1A9, UGT1A5*	rs887829	234668570	intron_variant	5.00E-25
5/9/2013	23371916	1,452 Han Chinese ancestry individuals	CHB	*UGT1A10, UGT1A8, UGT1A5, UGT1A3, UGT1A6, UGT1A9, UGT1A4, UGT1A7*	rs11891311	234639310	intron_variant	1.00E-41
5/9/2013	23371916	1,452 Han Chinese ancestry individuals	CHB	*UGT1A10, UGT1A6, UGT1A4, UGT1A8, UGT1A3, UGT1A1, UGT1A9, UGT1A7, UGT1A5*	rs4148323	234669144	missense_variant	5.00E-69

5/9/2013	23371916	1,452 Han Chinese ancestry individuals	CHB	*UGT1A10, UGT1A6, UGT1A4, UGT1A8, UGT1A3, UGT1A1, UGT1A9, UGT1A7, UGT1A5*	rs4148323	234669144	missense_variant	7.00E-30
5/9/2013	23371916	1,452 Han Chinese ancestry individuals	CHB	*UGT1A10, UGT1A6, UGT1A4, UGT1A8, UGT1A3, UGT1A1, UGT1A9, UGT1A7, UGT1A5*	rs4148323	234669144	missense_variant	2.00E-62
5/9/2013	23371916	1,452 Han Chinese ancestry individuals	CHB	*UGT1A6, UGT1A8, UGT1A1, UGT1A3, UGT1A9, UGT1A4, UGT1A5, UGT1A7, UGT1A10*	rs6742078	234672639	intron_variant	1.00E-89
5/9/2013	23371916	1,452 Han Chinese ancestry individuals	CHB	*UGT1A6, UGT1A8, UGT1A1, UGT1A3, UGT1A9, UGT1A4, UGT1A5, UGT1A7, UGT1A10*	rs6742078	234672639	intron_variant	6.00E-40
5/9/2013	23371916	1,452 Han Chinese ancestry individuals	CHB	*UGT1A6, UGT1A8, UGT1A1, UGT1A3, UGT1A9, UGT1A4, UGT1A5, UGT1A7, UGT1A10*	rs6742078	234672639	intron_variant	2.00E-78
9/7/2013	23642732	1,180 European ancestry individuals from ~475 families	CEU	*UGT1A6, UGT1A8, UGT1A7, UGT1A4, UGT1A10, UGT1A3, UGT1A9, UGT1A5*	rs887829	234668570	intron_variant	9.00E-20
6/11/2015	25246029	1,868 European ancestry cases	CEU	*UGT1A7, UGT1A6, UGT1A9, UGT1A1, UGT1A4, UGT1A10, UGT1A8, UGT1A5, UGT1A3*	rs4148325	234673309	intron_variant	5.00E-93
10/24/2015	25884002	2,547 individuals	CEU-YRI	*UGT1A7, UGT1A9, UGT1A8, UGT1A10*	rs10173355	234597321	intron_variant	5.00E-16
10/24/2015	25884002	2,547 individuals	CEU-YRI	*UGT1A6, AC114812.2, UGT1A8, UGT1A9, UGT1A10, UGT1A7*	rs17863787	234611094	intron_variant	1.00E-22
10/24/2015	25884002	2,547 individuals	CEU-YRI	*UGT1A6, UGT1A9, UGT1A8, UGT1A5, UGT1A10, UGT1A7*	rs1875263	234625622	intron_variant	7.00E-12
10/24/2015	25884002	2,547 individuals	CEU-YRI	*UGT1A12P - UGT1A11P*	rs2741012	234508963	regulatory_region_variant	2.00E-09
10/24/2015	25884002	2,547 individuals	CEU-YRI	*UGT1A3, UGT1A4, UGT1A5, UGT1A8, UGT1A7, UGT1A9, UGT1A6, UGT1A10*	rs3755319	234667582	intron_variant	9.00E-20

10/24/2015	25884002	2,547 individuals	CEU-YRI	*UGT1A7, UGT1A6, UGT1A9, UGT1A1, UGT1A4, UGT1A10, UGT1A8, UGT1A5, UGT1A3*	rs4148325	234673309	intron_variant	1.00E-30
10/24/2015	25884002	2,547 individuals	CEU-YRI	*UGT1A8, UGT1A6, UGT1A4, UGT1A5, UGT1A7, UGT1A3, UGT1A9, UGT1A10*	rs4477910	234643737	intron_variant	5.00E-16
10/24/2015	25884002	2,547 individuals	CEU-YRI	*UGT1A4, UGT1A9, UGT1A8, UGT1A7, UGT1A6, UGT1A10, UGT1A5, UGT1A3*	rs4663969	234655313	intron_variant	4.00E-16
10/24/2015	25884002	2,547 individuals	CEU-YRI	*UGT1A6, UGT1A8, UGT1A7, UGT1A4, UGT1A10, UGT1A3, UGT1A9, UGT1A5*	rs887829	234668570	intron_variant	7.00E-31
10/24/2015	25884002	2,547 individuals	CEU-YRI	*UGT1A10, UGT1A6, UGT1A7, UGT1A9, UGT1A4, UGT1A1, UGT1A3, UGT1A5, UGT1A8*	rs929596	234674476	intron_variant	3.00E-24
8/13/2018	26148204	243 European ancestry cases, 149 African American cases, 100 Hispanic American cases, 9 unknown ancestry cases	CEU-YRI	*UGT1A6, UGT1A4, UGT1A9, UGT1A8, UGT1A3, UGT1A7, UGT1A10, UGT1A5*	rs10178992	234657877	intron_variant	6.00E-09
8/13/2018	26148204	243 European ancestry cases, 149 African American cases, 100 Hispanic American cases, 9 unknown ancestry cases	CEU-YRI	*UGT1A6, UGT1A7, UGT1A4, UGT1A5, UGT1A3, UGT1A9, UGT1A8, UGT1A10*	rs10179091	234663649	intron_variant	1.00E-09
8/13/2018	26148204	243 European ancestry cases, 149 African American cases, 100 Hispanic American cases, 9 unknown ancestry cases	CEU-YRI	*UGT1A10, UGT1A5, UGT1A6, UGT1A7, UGT1A8, UGT1A9, UGT1A3, UGT1A4, AC114812.1*	rs10929301	234663649	splice_region_variant	7.00E-09
8/13/2018	26148204	243 European ancestry cases, 149 African American cases, 100 Hispanic American cases, 9 unknown ancestry cases	CEU-YRI	*UGT1A8, UGT1A9, UGT1A7, UGT1A6, UGT1A5, UGT1A10, UGT1A3, UGT1A4*	rs10929302	234665782	intron_variant	2.00E-09
8/13/2018	26148204	243 European ancestry cases, 149 African American cases, 100 Hispanic American cases, 9 unknown ancestry cases	CEU-YRI	*UGT1A6, UGT1A9, UGT1A8, UGT1A10, UGT1A3, UGT1A5, UGT1A7, UGT1A4*	rs11673726	234664060	non_coding_transcript_exon_variant	4.00E-09
8/13/2018	26148204	243 European ancestry cases, 149 African American cases, 100 Hispanic American cases, 9	CEU-YRI	*UGT1A3, UGT1A5, UGT1A8, UGT1A7, UGT1A6,*	rs11695484	234654449	intron_variant	3.00E-09

		unknown ancestry cases		*UGT1A10, UGT1A9, UGT1A4*				
8/13/2018	26148204	243 European ancestry cases, 149 African American cases, 100 Hispanic American cases, 9 unknown ancestry cases	CEU-YRI	*UGT1A5, UGT1A10, UGT1A2P, UGT1A4, UGT1A3, UGT1A7, UGT1A8, UGT1A9, UGT1A6*	rs11888459	234656640	non_coding_transcript_exon_variant	6.00E-09
8/13/2018	26148204	243 European ancestry cases, 149 African American cases, 100 Hispanic American cases, 9 unknown ancestry cases	CEU-YRI	*UGT1A6, UGT1A9, AC114812.3, UGT1A5, UGT1A7, UGT1A3, UGT1A4, UGT1A10, UGT1A8*	rs17862875	234649302	intron_variant	6.00E-09
8/13/2018	26148204	243 European ancestry cases, 149 African American cases, 100 Hispanic American cases, 9 unknown ancestry cases	CEU-YRI	*UGT1A7, UGT1A10, UGT1A8, UGT1A6, UGT1A9, UGT1A3, UGT1A5, UGT1A4*	rs17864701	234652717	intron_variant	5.00E-09
8/13/2018	26148204	243 European ancestry cases, 149 African American cases, 100 Hispanic American cases, 9 unknown ancestry cases	CEU-YRI	*UGT1A5, UGT1A7, UGT1A8, UGT1A4, UGT1A10, UGT1A6, UGT1A3, UGT1A9*	rs2885296	234659061	intron_variant	2.00E-09
8/13/2018	26148204	243 European ancestry cases, 149 African American cases, 100 Hispanic American cases, 9 unknown ancestry cases	CEU-YRI	*AC114812.3, UGT1A6, UGT1A8, UGT1A4, UGT1A5, UGT1A7, UGT1A9, UGT1A3, UGT1A10*	rs34352510	234650562	non_coding_transcript_exon_variant	3.00E-10
8/13/2018	26148204	243 European ancestry cases, 149 African American cases, 100 Hispanic American cases, 9 unknown ancestry cases	CEU-YRI	*UGT1A4, UGT1A8, UGT1A9, UGT1A1, UGT1A10, UGT1A3, UGT1A5, UGT1A6, UGT1A7*	rs3771341	234673239	intron_variant	1.00E-10
8/13/2018	26148204	243 European ancestry cases, 149 African American cases, 100 Hispanic American cases, 9 unknown ancestry cases	CEU-YRI	*UGT1A1, UGT1A8, UGT1A7, UGT1A9, UGT1A5, UGT1A6, UGT1A3, UGT1A10, UGT1A4*	rs4148324	234672722	intron_variant	9.00E-11
8/13/2018	26148204	243 European ancestry cases, 149 African American cases, 100 Hispanic American cases, 9 unknown ancestry cases	CEU-YRI	*UGT1A7, UGT1A6, UGT1A9, UGT1A1, UGT1A4, UGT1A10, UGT1A8, UGT1A5, UGT1A3*	rs4148325	234673309	intron_variant	1.00E-11
8/13/2018	26148204	243 European ancestry cases, 149 African American cases, 100 Hispanic American cases, 9 unknown ancestry cases	CEU-YRI	*UGT1A5, UGT1A8, UGT1A3, UGT1A9, UGT1A6, UGT1A10, UGT1A4, UGT1A7*	rs6714634	234664765	non_coding_transcript_exon_variant	2.00E-09
8/13/2018	26148204	243 European ancestry cases, 149 African American cases, 100 Hispanic American cases, 9	CEU-YRI	*UGT1A6, UGT1A8, UGT1A1, UGT1A3, UGT1A9,*	rs6742078	234672639	intron_variant	4.00E-11

		unknown ancestry cases		*UGT1A4, UGT1A5, UGT1A7, UGT1A10*				
8/13/2018	26148204	243 European ancestry cases, 149 African American cases, 100 Hispanic American cases, 9 unknown ancestry cases	CEU-YRI	*UGT1A10, UGT1A8, UGT1A4, UGT1A6, UGT1A9, UGT1A3, UGT1A5, UGT1A7*	rs6747843	234664354	non_coding_transcript_exon_variant	2.00E-09
8/13/2018	26148204	243 European ancestry cases, 149 African American cases, 100 Hispanic American cases, 9 unknown ancestry cases	CEU-YRI	*UGT1A4, UGT1A10, UGT1A6, UGT1A5, UGT1A9, UGT1A7, UGT1A8, UGT1A3*	rs7604115	234658116	intron_variant	6.00E-09
8/13/2018	26148204	243 European ancestry cases, 149 African American cases, 100 Hispanic American cases, 9 unknown ancestry cases	CEU-YRI	*UGT1A10, UGT1A6, UGT1A7, UGT1A9, UGT1A4, UGT1A1, UGT1A3, UGT1A5, UGT1A8*	rs929596	234674476	intron_variant	1.00E-10
7/28/2018	29403010	110,207 Japanese ancestry individuals	CHB	*UGT1A5, UGT1A10, UGT1A7, UGT1A4, UGT1A8, UGT1A9, UGT1A6*	rs13401281	234628679	intron_variant	1E-718
7/28/2018	29403010	110,207 Japanese ancestry individuals	CHB	*UGT1A10, UGT1A5, UGT1A9, UGT1A3, UGT1A4, UGT1A6, UGT1A8, UGT1A7*	rs28898617	234637789	missense_variant	1.00E-10
7/28/2018	29403010	110,207 Japanese ancestry individuals	CHB	*UGT1A9, UGT1A8, UGT1A5, UGT1A6, UGT1A10, UGT1A1, UGT1A4, UGT1A3, UGT1A7*	rs28946889	234671462	intron_variant	3E-1255
7/28/2018	29403010	110,207 Japanese ancestry individuals	CHB	*UGT1A5, UGT1A6, UGT1A7, UGT1A8, UGT1A9, UGT1A10, UGT1A4, UGT1A3, UGT1A1*	rs6431630	234677386	intron_variant	3.00E-81
7/28/2018	29403010	110,207 Japanese ancestry individuals	CHB	*UGT1A5, UGT1A7, UGT1A8, UGT1A9, UGT1A3, UGT1A4, UGT1A6, UGT1A10*	rs75520741	234641604	intron_variant	3E-690
7/28/2018	29403010	110,207 Japanese ancestry individuals	CHB	*UGT1A7, UGT1A4, UGT1A1, UGT1A6, UGT1A3, UGT1A9, UGT1A8, UGT1A5, UGT1A10*	rs8330	234681645	3_prime_UTR_variant	4.00E-20
2/1/2019	30621171	188 European ancestry male individuals, 242 European ancestry female individuals	CEU	*UGT1A6, UGT1A10, AC114812.2, UGT1A8, UGT1A9, UGT1A7*	rs12988520	234607394	intron_variant	5.00E-09

2/1/2019	30621171	188 European ancestry male individuals, 242 European ancestry female individuals	CEU	*UGT1A6, AC114812.2, UGT1A8, UGT1A9, UGT1A10, UGT1A7*	rs17863787	234611094	intron_variant	1.00E-17
2/1/2019	30621171	188 European ancestry male individuals, 242 European ancestry female individuals	CEU	*UGT1A7, UGT1A6, UGT1A9, UGT1A10, AC114812.2, UGT1A8*	rs2070959	234602191	missense_variant	2.00E-14
2/1/2019	30621171	188 European ancestry male individuals, 242 European ancestry female individuals	CEU	*UGT1A7, UGT1A6, UGT1A9, UGT1A1, UGT1A4, UGT1A10, UGT1A8, UGT1A5, UGT1A3*	rs4148325	234673309	intron_variant	2.00E-19
2/1/2019	30621171	188 European ancestry male individuals, 242 European ancestry female individuals	CEU	*UGT1A10, UGT1A6, UGT1A7, UGT1A9, UGT1A4, UGT1A1, UGT1A3, UGT1A5, UGT1A8*	rs929596	234674476	intron_variant	8.00E-15

* March 2019 GWAS Catalog
